# Realizing Intrinsically Ultralow and Glass‐Like Thermal Transport via Chemical Bonding Engineering

**DOI:** 10.1002/advs.202417292

**Published:** 2025-03-07

**Authors:** Zhonghao Xia, Xingchen Shen, Jun Zhou, Yuling Huang, Yali Yang, Jiangang He, Yi Xia

**Affiliations:** ^1^ Key Laboratory of Advanced Materials and Devices for Post‐Moore Chips Ministry of Education School of Mathematics and Physics University of Science and Technology Beijing Beijing 100083 China; ^2^ CRISMAT, CNRS ENSICAEN Caen 14000 France; ^3^ MOE Key Laboratory of Material Physics and Chemistry Under Extraordinary Conditions School of Physical Science and Technology Northwestern Polytechnical University Xi'an 710072 People's Republic of China; ^4^ Basic Experimental Center for Natural Science University of Science and Technology Beijing Beijing 100083 China; ^5^ Department of Mechanical and Energy Engineering Southern University of Science and Technology (SUSTech) Shenzhen 518055 China; ^6^ Department of Mechanical & Materials Engineering Portland State University Portland OR 97201 USA

**Keywords:** chemical bonding principles, lattice thermal conductivity, material design and discovery, solid state chemistry

## Abstract

Crystals exhibiting glass‐like and low lattice thermal conductivity ($\kappa_{L}$) are not only scientifically intriguing but also practically valuable in various applications, including thermal barrier coatings, thermoelectric energy conversion, and thermal management. However, such unusual $\kappa_{L}$ are typically observed only in compounds containing heavy elements, with large unit cells, or at high temperatures. In this study, chemical bonding principles are utilized to weaken the Ag–Ag bonds and enhance lattice anharmonicity. The incorporation of a chalcogen anion as a bridge ligand is proposed to facilitate phonon rattling in Ag_6_‐octahedron‐based compounds. Guided by this design strategy, five Ag_6_ octahedron‐based compounds, *A*Ag_3_ *X*_2_ (*A* = Li, Na, and K; *X* = S and Se), which are characterized by low average atomic masses and exhibit exceptionally strong four‐phonon scattering, are theoretically identified. Consequently, these compounds demonstrate ultralow thermal conductivities (0.3–0.6 W m^−1^ K^−1^) with minimal temperature dependence (T^−0.1^) across a wide temperature range. Experimental validation confirms that the κ_L_ of NaAg_3_S_2_ is 0.45 W m^−1^ K^−1^ within the temperature range of 200–550 K. The results clearly demonstrate that weak chemical bonding plays a crucial role in designing compounds with glass‐like κ_L_, highlighting the effectiveness of chemical bonding engineering in achieving desired thermal transport properties.

## Graphical abstract

Lattice thermal conductivity can be effectively suppressed by introducing a S^2 −^ anion into the bridging position between the Ag–Ag bond in the Ag_6_ octahedron, which weakens the Ag–Ag bond within the octahedral structure and enhances phonon‐phonon scattering rates.

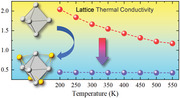

## 1 Introduction

Lattice thermal conductivity ($\kappa_{L}$) is a fundamental property of solids, and crystalline dielectric materials exhibiting extreme $\kappa_{L}$ values are essential for various technological applications, including thermoelectrics, thermal management, and thermal barrier coatings.^[^ ^1^, ^2^, ^3^ ^]^ Within the framework of the phonon gas model,^[^ ^4^ ^]^ heat carriers are identified as propagating phonons, while heat resistance primarily arises from intrinsic phonon–phonon scattering. At temperatures exceeding the Debye temperature, Umklapp phonon–phonon interactions generally dominate the scattering processes.^[^ ^5^ ^]^ As a result, $\kappa_{L}$ typically decreases rapidly with increasing temperature, often following the relationship $\kappa_{L}$ ∝ T^−1^.^[^ ^6^ ^]^ However, certain materials deviate from this T^−1^ behavior, exhibiting temperature‐independent $\kappa_{L}$ under specific conditions, which reflects glass‐like thermal conductivity characteristics. While low and temperature‐independent $\kappa_{L}$ is a defining feature of amorphous materials,^[^ ^7^ ^]^ it is very rarely observed in certain crystalline solids, primarily due to the lack of periodicity in amorphous phases.^[^ ^3^, ^8^, ^9^ ^]^

It is noteworthy that glass‐like thermal conductivities have occasionally been observed in crystalline solids, particularly when the structures are complex, or when impurities and disordered atoms are present, or at sufficiently high temperatures.^[^ ^9^, ^10^ ^]^ Compounds exemplifying partial atomic disorder include zinc‐disordered β‐Zn_4_Sb_3_,^[^ ^11^ ^]^ liquid‐like Cu_2−*x*_Se,^[^ ^12^ ^]^ and Cu‐ and Ag‐based argyrodites.^[^ ^13^, ^14^, ^15^ ^]^ In the case of β‐Zn_4_Sb_3_, Zn interstitials exhibit significant mobilities, diffusing throughout the structure and leading to glass‐like thermal conductivity.^[^ ^11^, ^16^ ^]^ In the high‐temperature phase (β) Cu_2−*x*_Se, the highly disordered Cu atoms surrounding the Se sublattice are superionic and possess liquid‐like mobility, contributing to the observed glass‐like thermal conductivity.^[^ ^12^ ^]^ The ultralow thermal transport in crystalline argyrodite Cu_7_PS_6_ is attributed to the softening of phonon modes, arising from disorder in the molten copper sublattice.^[^ ^13^, ^14^ ^]^ Since anharmonicity typically increases with temperature, the glass‐like thermal conductivity is expected at high enough temperature, as long as the lattice is not melted. For example, La_2_Zr_2_O_7_ exhibits crystal‐like thermal conductivity at room temperature but glass‐like thermal conductivity at the temperature higher than 900 K.^[^ ^17^ ^]^

To date, only a limited number of crystalline solids devoid of impurities and disorder exhibit glass‐like $\kappa_{L}$. Most of these materials are layered compounds or systems characterized by large and complex unit cells, such as clathrates and skutterudites.^[^ ^18^, ^19^ ^]^ In these cage‐like structures, the relative sizes of the guest atoms and the cages are crucial factors influencing whether a specific composition manifests glass‐like or crystal‐like $\kappa_{L}$. This relationship is linked to the displacement amplitudes of the guest atoms, which in turn affects the strength of anharmonic phonon–phonon scattering. Notably, layered halide perovskites, such as Cs_3_Bi_2_Br_9_ and Cs_3_Bi_2_I_6_Cl_3_, have been found to exhibit extremely low glass‐like thermal conductivities,^[^ ^20^, ^21^ ^]^ attributable to their heavy atomic mass, low acoustic phonon frequencies and sound velocities.

Interestingly, Cu_12_Sb_4_S_13_, which exhibits neither a cage‐like nor a layered structure, demonstrates an exceptionally low $\kappa_{L}$ with glass‐like behavior ranging from 200 to 600 K.^[^ ^22^, ^23^ ^]^ A previous theoretical study revealed that the temperature‐induced hardening of low‐lying optical modes effectively mitigates the scattering of heat‐carrying acoustic modes, which is otherwise exacerbated by the rattling of Cu cations within the CuS_3_ triangle. This mitigation occurs through the reduction of available phase space for three‐phonon processes, thereby counterbalancing the conventional ∝ T increase in scattering associated with phonon population and resulting in a nearly temperature‐independent $\kappa_{L}$.^[^ ^24^ ^]^ The rattling of Cu within the CuS_3_ triangle can be attributed to the *p*‐*d* antibonding interactions between the coinage metal cation and the chalcogenide anion,^[^ ^25^ ^]^ which leads to chemical bond softening and atomic rattling. Similarly, the observed low acoustic phonon frequencies and sound velocities in Cs_3_Bi_2_Br_9_ and Cs_3_Bi_2_I_6_Cl_3_ can also be ascribed to the weak bonding interactions between Bi and the halide cations, alongside their substantial atomic masses. These findings suggest that weak chemical bonds may offer a promising avenue for the design and discovery of compounds characterized by glass‐like $\kappa_{L}$. Additionally, bonding inhomogeneity strategy has been identified as a contributing factor to the ultralow κ_L_ observed in a number of compounds.^[^ ^26^, ^27^, ^28^, ^29^ ^]^

In this study, we propose a strategy for designing crystalline solids with glass‐like $\kappa_{L}$ based on chemical bonding principles. Guided by this design strategy, we identified five Ag_6_ octahedron‐based semiconductors that exhibit exceptionally low $\kappa_{L}$ values and glass‐like thermal conductivities within the temperature range of 200–550 K, using first‐principles calculations. We experimentally validated our predictions for NaAg_3_S_2_. Our results, derived from state‐of‐the‐art calculations and experimental investigations, demonstrate that NaAg_3_S_2_ possesses a $\kappa_{L}$ of 0.45 W m^−1^ K^−1^ across the temperature range of 200–550 K. This behavior is attributed to the interplay between flat‐band phonon hardening and increased three‐ and four‐phonon scattering rates with rising temperature, which arise from the antibonding interactions between Ag and S, as well as the weak chemical bonding between Ag atoms in Ag_6_ octahedron. Furthermore, our calculations indicate that KAg_3_S_2_ exhibits a similar thermal conductivity behavior, with an even lower $\kappa_{L}$ of 0.23 W m^−1^ K^−1^. The rattling motion of Ag atoms disrupts the conventional phonon‐gas model, underscoring the significant role that wave‐like (phonon tunneling) thermal conductivity plays in shaping $\kappa_{L}$.

## 2 Results and Discussion

### 2.1 Materials Design Strategies and New Materials Discovery

Previous studies indicate that Ag_3_Ge_5_P_6_ exhibits low $\kappa_{L}$ of 1.7 W m^−1^ K^−1^ at 300 K, with a temperature dependence of 1/T^0.59^ ranging from 200 to 600 K.^[^ ^30^, ^31^ ^]^ This anomalous behavior in lattice heat transport has been primarily attributed to the presence of flat phonon bands, predominantly involving Ag_6_ octahedra.^[^ ^32^ ^]^ This is further evidenced by the large mean square atomic displacements (MSD) of Ag atoms and the hardening of the low‐lying flat phonon bands as the temperature increases.^[^ ^31^, ^32^ ^]^ It has been established that the weak Ag‐Ag bond plays a significant role in scattering heat‐carrying acoustic phonons, thereby contributing to the unusual heat transfer behavior.^[^ ^32^ ^]^ The weakness of the Ag–Ag bond arises from the hybridization of the *d* orbitals with the *s* and *p* orbitals of Ag^+^ cation.^[^ ^33^ ^]^ This observation prompts crucial follow‐up questions: Is it possible to further weaken the Ag‐Ag bond? Could such modifications potentially result in a lower $\kappa_{L}$ and a flatter temperature dependence of the $\kappa_{L}$ ‐ T curve?

In 1990, Cui and Kertesz demonstrated that the presence of a bridge ligand *X* between two metal *M* ions with a *d* ^10^ electron configuration significantly reduces the *M*–*M* bonding interaction compared to a *M*–*M* bond without the bridge ligand *X*.^[^ ^34^ ^]^ This reduction is attributed to the filling of the antibonding states formed by the *d* orbitals of *M* and the *p* orbitals of *X*. Based on this finding, it is anticipated that introducing a bridge ligand *X* in the Ag–Ag bond of an Ag_6_ octahedron could weaken the Ag–Ag interactions further, potentially leading to enhanced anharmonicity and a lower $\kappa_{L}$. Guided by this design principle, we conducted a search for suitable compounds within the silver chalcogen chemical space using the Inorganic Crystal Structure Database (ICSD).^[^ ^35^ ^]^ The objective was to identify materials that exhibit low $\kappa_{L}$ with reduced temperature dependence.

**Figure 1:**
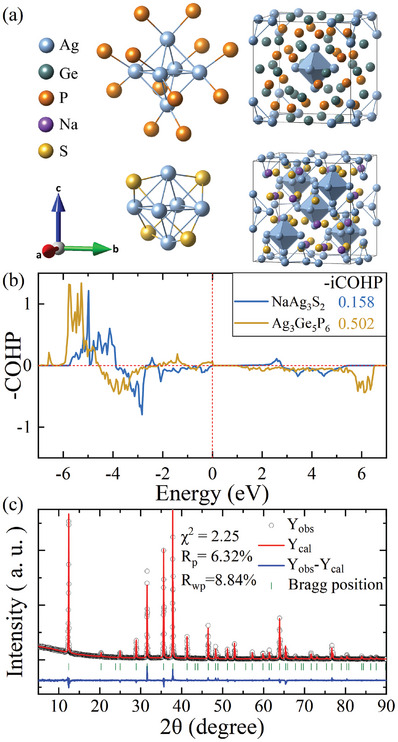
a) The left panel is Ag_6_ octahedral and S or P atom bonding modes, and the right panel is the crystal structure of NaAg_3_S_2_ and Ag_3_Ge_5_P_6_, wherein an Ag_6_ octahedral cluster is highlighted. b) ‐COHP and ‐iCOHP of the Ag–Ag bond in the Ag_6_ octahedron of NaAg_3_S_2_ ang Ag_3_Ge_5_P_6_. c) The refined PXRD of the hot‐pressed powder NaAg_3_S_2_ sample at 300 K.

We identified two experimentally synthesized and isostructural compounds, NaAg_3_S_2_ and KAg_3_S_2_, that feature Ag_6_ octahedra with S^2 −^ anions equally bonding to three Ag^+^ cations on one face of the octahedron (see **Figure** **1**). NaAg_3_S_2_ was first synthesized by Huster et al.,^[^ ^36^ ^]^ while KAg_3_S_2_ was introduced by Wood et al.^[^ ^37^ ^]^ Both compounds crystallize in the cubic system with the space group $Fd\bar{3}m$ (No. 227). The Na^+^/K^+^, Ag^+^, and S^2 −^ ions occupy the 16*c*, 48*f*, and 32*e* Wyckoff positions, respectively, with site symmetries of $\bar{3}m$, 2mm, and 3m. In these structures, Na^+^/K^+^ ions are located at the center of the octahedron formed by S^2 −^ anions, while six Ag^+^ cations configure into a regular Ag_6_ octahedron, with Ag‐Ag bonds constituting the edges. The NaS_6_ octahedra are interconnected through edge‐ and face‐sharing, creating a 3D network. This structure can be viewed as the embedding of Ag_6_ octahedra within the framework formed by edge‐sharing NaS_6_ octahedra. At each face of the Ag_6_ octahedra, an S^2 −^ anion bonds equally to three Ag^+^ cations. The bond length between the S^2 −^ anion and the Ag^+^ cation is measured at 2.4418 Å for NaAg_3_S_2_ and 2.4163 Å for KAg_3_S_2_, closely aligning with the Ag–S bond length of 2.3881 Å calculated using PBEsol for α‐Ag_2_S, indicating a robust bonding interaction between Ag^+^ and S^2 −^. In contrast, the Ag–Ag bond lengths in NaAg_3_S_2_ (2.9760 Å) and KAg_3_S_2_ (2.9561 Å) are significantly greater than those found in Ag_3_Ge_5_P_6_ (2.8426 Å) and the bulk silver (2.88 Å).^[^ ^34^ ^]^ This elongation is attributed to the presence of the bridging S^2 −^ anion between the Ag–Ag bonds.^[^ ^38^ ^]^

**Figure 2:**
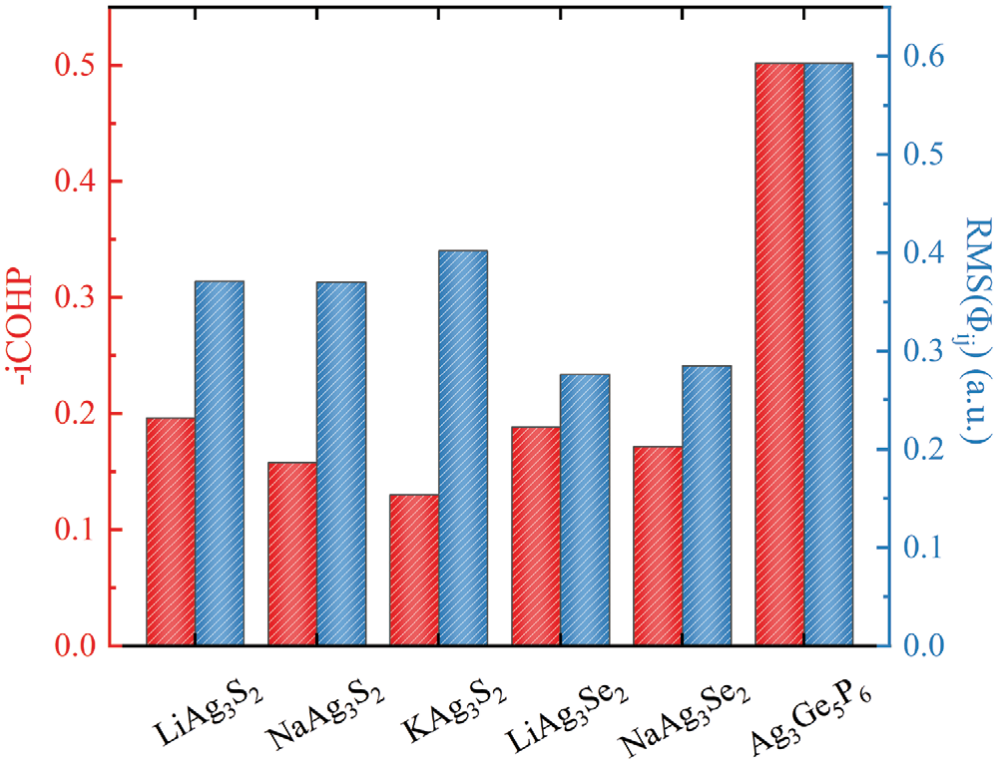
The Ag–Ag bond ‐iCOHP and Φ_*ij*_ of Ag_3_Ge_5_P_6_ and NaAg_3_S_2_‐type compounds.

The crystal orbital Hamilton population (COHP) and integrated COHP (iCOHP) of NaAg_3_S_2_ and Ag_3_Ge_5_P_6_ are presented in Figure 1b. The Ag–Ag bond of NaAg_3_S_2_ exhibits stronger antibonding states than that of Ag_3_Ge_5_P_6_ in the energy range from –5 eV to the Fermi level. Therefore, the ‐iCOHP of NaAg_3_S_2_ is much smaller than that of Ag_3_Ge_5_P_6_. The Ag–Ag bond lengths and the ‐iCOHP of Ag_3_Ge_5_P_6_, NaAg_3_S_2_, and KAg_3_S_2_ are depicted in **Figure** **2** and summarized in Table S1 (Supporting Information). Notably, the Ag–Ag bond lengths in all NaAg_3_S_2_‐type compounds are indeed longer than that in Ag_3_Ge_5_P_6_, and the ‐iCOHP values for the Ag–Ag bonds in NaAg_3_S_2_‐type compounds are substantially lower than that in Ag_3_Ge_5_P_6_, indicating significantly weaker Ag–Ag bonds in the former two compounds.

We conducted a further search for thermodynamically stable NaAg_3_S_2_‐type compounds within the family of *A*Ag_3_ *X*_2_ (*A* = Li, Na, K, Rb, Cs, and *X* = S or Se) compounds through prototype structure decoration. Notably, the compounds LiAg_3_S_2_, LiAg_3_Se_2_, and NaAg_3_Se_2_ have not yet been experimentally reported, while KAg_3_Se_2_, RbAg_3_S_2_, RbAg_3_Se_2_, CsAg_3_S_2_, and CsAg_3_Se_2_ crystallize in the CsAg_3_S_2_‐type structure (space group C2/m, No. 12). Thus, we first compared the total energies of the NaAg_3_S_2_‐type and CsAg_3_S_2_‐type structures for all *A*Ag_3_ *X*_2_ compounds. As illustrated in Figure S1 (Supporting Information), LiAg_3_S_2_, NaAg_3_S_2_, and NaAg_3_Se_2_ exhibit a preference for the NaAg_3_S_2_‐type structure, while the remaining compounds favor the CsAg_3_S_2_‐type structure. Moreover, the energy difference between these two structures increases linearly with the ionic radius of *A*, suggesting that compounds with larger *A* ions are more inclined to adopt the CsAg_3_S_2_‐type structure over the NaAg_3_S_2_‐type structure. This implies that the experimentally observed $Fd\bar{3}m$ phase of KAg_3_S_2_ is a metastable phase. Convex hull calculations indicate that LiAg_3_Se_2_, NaAg_3_S_2_, and NaAg_3_Se_2_ are thermodynamically stable (i.e., they lie on the convex hull), whereas LiAg_3_S_2_ and KAg_3_S_2_ are metastable (i.e., positioned above the convex hull with a convex hull distance of less than 50 meV atom^−1^) at 0 K, when assessed against their competing phases, as determined by the OQMD,^[^ ^39^, ^40^ ^]^ as shown in Table S1 (Supporting Information). Similar to NaAg_3_S_2_ and KAg_3_S_2_, the Ag–Ag bond lengths and ‐iCOHP for all other NaAg_3_S_2_‐type compounds are significantly smaller than those of Ag_3_Ge_5_P_6_, as depicted in Figure 2 and Table S1 (Supporting Information). Our calculations of elastic constant and molecular dynamic simulations indicate that these compounds are mechanically stable, see Table S5 and Figure S8 (Supporting Information). Additionally, we compared the second‐order interatomic force constants (2ndIFC) for the Ag–Ag bond, which are directly correlated to vibrational frequencies. To quantify the magnitude of these force constants, we utilized the root mean square (RMS) of the elements of the 2ndIFC tensor (Φ_*ij*_).^[^ ^41^ ^]^ It is evident that all NaAg_3_S_2_‐type compounds exhibit a significantly smaller RMS value of Φ_*ij*_ for the Ag–Ag bond compared to that of Ag_3_Ge_5_P_6_.

### 2.2 Phonon Dispersion at 0 and 300 K

**Figure 3:**
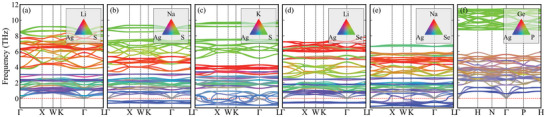
(a–f) are the phonon dispersion of LiAg_3_S_2_, NaAg_3_S_2_, KAg_3_S_2_, LiAg_3_Se_2_, NaAg_3_Se_2_, and Ag_3_Ge_5_P_6_ at 0 K, respectively. Red, blue, and green colors represent the contributions of *A*, Ag, and *X* (P) atoms to the phonon branches, respectively.

The phonon dispersion and $\kappa_{L}$ of LiAg_3_S_2_, LiAg_3_Se_2_, NaAg_3_S_2_, NaAg_3_Se_2_, KAg_3_S_2_, and Ag_3_Ge_5_P_6_ were computed using a state‐of‐the‐art calculation method,^[^ ^42^, ^43^, ^44^ ^]^ which accounts for three‐ and four‐phonon interactions. This computational approach elucidates the effects of Ag–Ag bonding on phonon dispersion and $\kappa_{L}$ by comparing five NaAg_3_S_2_‐type compounds with Ag_3_Ge_5_P_6_. The phonon dispersion at 0 K for the NaAg_3_S_2_‐type compounds and Ag_3_Ge_5_P_6_, calculated using the 2ndIFC, is presented in **Figure** **3**. All of these compounds exhibit low‐frequency and flat‐band phonons primarily associated with Ag atoms. Flat‐band phonons indicate highly localized atomic vibrations and are characteristic of rattling phonon modes, which are typically observed in compounds that contain weakly bonded single or multiple atoms.^[^ ^32^, ^45^, ^46^, ^47^, ^48^ ^]^ The atomic vibrations corresponding to these flat‐band phonon modes at the Γ point of the Brillouin zone are illustrated in Figure S2 (Supporting Information). It is noteworthy that the vibrations of the atoms in both NaAg_3_S_2_ and Ag_3_Ge_5_P_6_ are predominantly confined to the Ag atoms. In the Ag_6_ octahedron, only three out of six Ag atoms participate in rotation, forming a head‐to‐tail triangle that is stretching in coordination with the remaining three Ag atoms. Consequently, the stiffness of this stretching mode, which determines the frequency of the phonon mode, is reliant on the strength of the Ag–Ag bond. Specifically, a weaker Ag–Ag bond strength results in a lower phonon frequency and a larger vibrational amplitude. The fourth and fifth phonon branches vibrate in a similar pattern, but in the opposite direction. These vibrational characteristics suggest that these Ag atoms behave as rattling phonon modes, akin to the behavior of a single atom within the cage‐like structures. In addition, the low‐lying and flat‐band phonon modes of Ag_3_Ge_5_P_6_ closely resemble the rattling modes observed in NaAg_3_S_2_.

**Figure 4:**
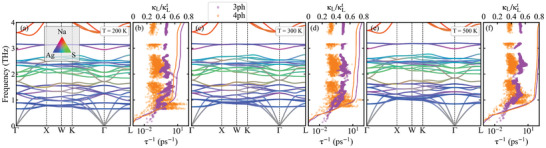
The phonon dispersion, 3ph‐ and 4ph‐scattering, and the relative cumulative $\kappa_{L}$ (the ratio of the cumulative $\kappa_{L}$ to total $\kappa_{L}$) of NaAg_3_S_2_ at 200, 300, and 500 K, respectively. Red, blue, and green colors represent the atomic contributions of Na, Ag, and S to each phonon band, respectively.

Furthermore, as depicted in Figure S3 (Supporting Information), Debye–Einstein fitting analysis of the low‐temperature specific heat capacity (C_*p*_) revealed the presence of two Einstein modes: $\Theta_{E_{1}}$ = 30 K (≈0.63 THz) and $\Theta_{E_{2}}$ = 65.4 K (≈1.35 THz). Notably, the low‐energy mode $\Theta_{E_{1}}$ aligns well with the theoretically predicted low‐lying optical phonons at ≈0.73 THz (see **Figure** **4**c), thereby validating our computational results. This correlation explains why the flat‐band phonon frequency of NaAg_3_S_2_ is lower than that of Ag_3_Ge_5_P_6_. As the size of *A* transitions from Li to K, the frequencies of the low‐frequency flat‐band phonons in the *A*Ag_3_ *X*_2_ compounds decrease, and more flat‐band phonons become unstable. This behavior is primarily attributed to the strain induced by the expansion of the *A*‐*X* bond length, rendering the NaAg_3_S_2_‐type structure less favorable for larger *A* cations, as discussed previously. The differences between *A*Ag_3_S_2_ and *A*Ag_3_Se_2_ can be traced to two factors: the variation in the anion radius and the bonding interaction difference between Ag and S and Ag and Se. These differences are rooted in the energy levels of the *p*‐orbitals in the chalcogen anions, which influence the overlap between the Ag *d* orbitals and the *p* orbitals of the ions.^[^ ^49^, ^50^ ^]^ Consequently, the *A*Ag_3_Se_2_ compounds exhibit smaller RMS values of Φ_*ij*_ compared to the *A*Ag_3_S_2_ compounds. Therefore, it is feasible to manipulate phonon behavior and heat transport by substituting the *A* and *X* ions.

**Figure 5:**
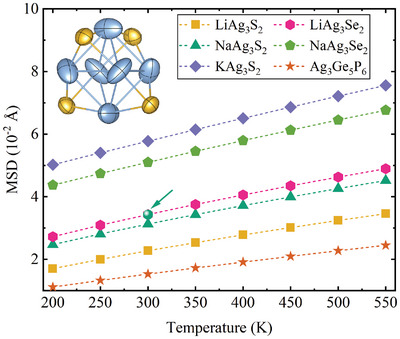
The calculated MSD of Ag atom as a function of temperature. The green circle highlighted by a green arrow shows the experimental value of NaAg_3_S_2_. The inset depicts the anisotropic thermal displacement ellipsoids of Ag and S atoms at 300 K.

The effect of temperature on phonon dispersion is investigated using self‐consistent phonon (SCPH) theory,^[^ ^42^, ^43^, ^44^ ^]^ which incorporates the first‐order corrections from fourth‐order force constants.^[^ ^43^, ^44^ ^]^ The temperature‐dependent phonon dispersion of NaAg_3_S_2_ across a range of 200–500 K is illustrated in Figure 4. It is evident that temperature significantly influences the low‐frequency phonon bands, particularly the flat‐band phonons, while having negligible effects on the remaining phonon bands, especially the high‐frequency ones. As the temperature increases, the frequencies of the flat‐band phonons exhibit a rapid hardening, whereas their dispersion remains relatively constant, indicating a strong temperature dependence of the 2ndIFC for Ag atoms. This phenomenon is further reflected in the MSD of the constituent atoms, which can be calculated from the renormalized second‐order force constants to account for temperature effects. As shown in **Figure** **5**, the magnitude of the Ag MSD increases linearly with rising temperature. Our calculated MSD values for NaAg_3_S_2_ align well with experimental observations. Additionally, all NaAg_3_S_2_‐type compounds examined in this study exhibit larger Ag MSD values compared to Ag_3_Ge_5_P_6_, consistent with our previous analysis that the NaAg_3_S_2_‐type structure possesses significantly weaker Ag–Ag bonding. The anisotropic thermal displacement ellipsoids of Ag can be attributed to its unique coordination environment. Specifically, the interactions between Ag‐S/Se and Ag–Ag bonds facilitate displacement that is more likely to occur in a direction perpendicular to the plane formed by Ag and its two nearest neighbor S/Se anions.

### 2.3 Lattice Thermal Conductivity

**Figure 6:**
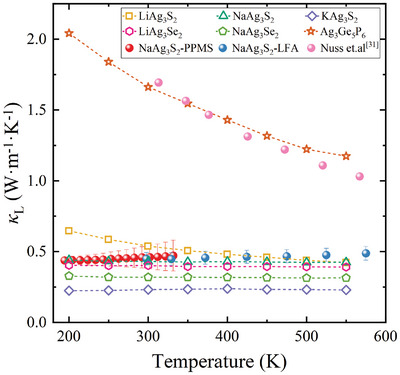
Experimental and computational $\kappa_{L}$ of *A*Ag_3_ *X*_2_ (*A* = Li, Na, and K; *X* = S and Se) and Ag_3_Ge_5_P_6_ as a function of temperature. The experimental $\kappa_{L}$ of Ag_3_Ge_5_P_6_ is taken from the single crystal data in Ref. [31].

**Figure 7:**
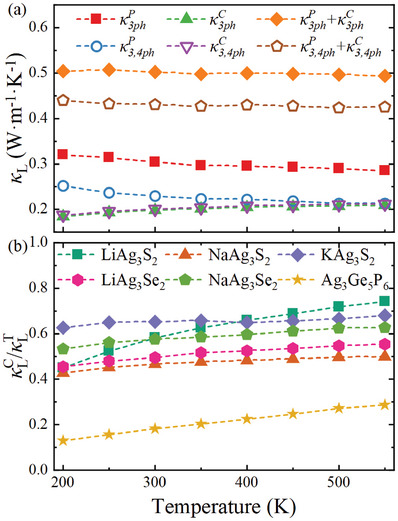
a) The compound NaAg_3_S_2_ temperature‐dependent $\kappa_{L}$ with the contribution from phonon populations and coherences in the framework of three‐ and four‐phonon scattering. b) The ratio of $\kappa_{L}$ from coherent contribution ($\kappa_{L}^{C}$) to total κ_L_ ($\kappa_{L}^{T}=\kappa_{L}^{P}+\kappa_{L}^{C}$) for all the studied compounds.

**Figure** **6** presents the calculated $\kappa_{L}$ for NaAg_3_S_2_‐type compounds alongside that of Ag_3_Ge_5_P_6_, employing a consistent computational methodology, see Experimental Section. The $\kappa_{L}$ value for Ag_3_Ge_5_P_6_ obtained from our calculations aligns well with experimental measurements, exhibiting a weak dependence on temperature, characterized by T^−0.59^. Notably, all NaAg_3_S_2_‐type compounds display considerably lower $\kappa_{L}$ values and a weaker temperature dependence compared to Ag_3_Ge_5_P_6_. Furthermore, the $\kappa_{L}$ of the NaAg_3_S_2_‐type compounds decreases with increasing atomic number of both *A* and *X* as one moves from S to Se. Among these compounds, KAg_3_S_2_ exhibits the lowest $\kappa_{L}$ at 0.23 W m^−1^ K^−1^ over the temperature range of 200–550 K, while LiAg_3_S_2_ displays the highest $\kappa_{L}$ value at 0.54 W m^−1^ K^−1^ at 300 K. It is important to note that our calculated $\kappa_{L}$ encompasses contributions from both particle‐like heat transport (the diagonal component of the heat flux operator, denoted as $\kappa_{L}^{P}$) and wave‐like interband tunneling (the off‐diagonal component of the heat flux operator, designated as $\kappa_{L}^{C}$).^[^ ^8^ ^]^ Our findings indicate that the off‐diagonal component makes a relatively larger contribution to the total $\kappa_{L}$ in the NaAg_3_S_2_‐type compounds, as depicted in **Figure** **7**. This observation is consistent with the enhanced anharmonicity associated with the weaker Ag–Ag and Ag‐S bonds present in these compounds.

The notably low $\kappa_{L}$ can be attributed to strong three‐phonon and four‐phonon scattering processes, as illustrated in Figure 4. The relative contribution of accumulated $\kappa_{L}$ (expressed as $\kappa_{L}\kappa_{L}^{t}$, where $\kappa_{L}^{t}$ denotes the total $\kappa_{L}$ reveals that phonons with frequencies below 2 THz account for 60% of the total $\kappa_{L}$, with the most significant contributions originating from the flat‐band phonons at ≈1 THz, where both three‐ and four‐phonon scattering processes play crucial roles. In this low‐frequency region (≈1 THz), the contribution from four‐phonon scattering becomes significant, coinciding with a notable overlap of acoustic and optical modes. In this context, the intensity of four‐phonon scattering may exceed that of three‐phonon scattering, leading to a pronounced reduction in $\kappa_{L}$. These flat branches near 1 THz possess smaller group velocities (ν_g_) and larger scattering phase spaces, contributing to the observed decrease in $\kappa_{L}$. As the temperature rises, the frequency of the flat‐band phonons increases, and the four‐phonon scattering rate experiences a slight decline, resulting in a gradual decrease in $\kappa_{L}^{P}$, as shown in Figure 7.

To verify our predictions, we experimentally synthesized NaAg_3_S_2_ polycrystals and measured its $\kappa_{L}$ from 200 to 550 K using both physical property measurement system (PPMS) and laser flash analysis (LFA) methods, with additional experimental details provided in the Methods section. As shown in Figure S6 (Supporting Information), our Differential Scanning Calorimetry (DSC) and Thermogravimetric Analysis (TGA) results demonstrate that NaAg_3_S_2_ exhibits good thermal stability within the temperature range of 300–600 K. Given the very high electrical resistance of NaAg_3_S_2_, the contribution of electrons to thermal conductivity is ignored in this work. As illustrated in Figure 6, the $\kappa_{L}$ of NaAg_3_S_2_ exhibits nearly temperature‐independent behavior over the range of 200 to 550 K, maintaining a value of ≈0.45 W m^−1^ K^−1^, indicative of glass‐like behavior. Notably, our calculated $\kappa_{L}$ for NaAg_3_S_2_ aligns very closely with the experimental measurements, not only in terms of the absolute values at specific temperatures but also concerning the temperature dependence observed from 200 to 550 K. Note the calculation of $\kappa_{L}$ becomes unphysical below 200 K due to the emergence of imaginary frequencies in this temperature regime. The minimum $\kappa_{L}$ of NaAg_3_S_2_, calculated using the diffusion model, is presented in Table S5 (Supporting information). Although NaAg_3_S_2_ exhibits ultralow, glass‐like κ_L_, our analysis indicates that there is potential for further reduction by introducing defects and engineered microstructures, such as the incorporation of dense dislocations, as demonstrated in BiCuSeO.^[^ ^51^ ^]^

### 2.4 Unique Attributes of NaAg_3_S_2_‐Type Compounds

**Figure 8:**
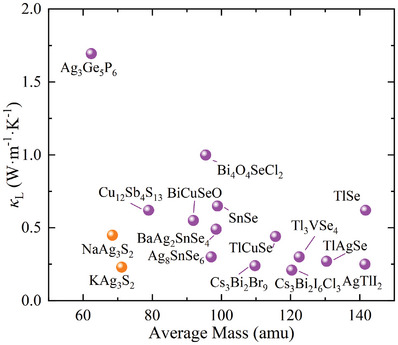
The dependence of κ_L_ at 300 K on averaged mass ($\bar{M}$). The purple balls come from the experimental values measured at room temperature in the refs. [20, 21, 22, 31, 52, 54, 55, 56, 57, 58, 59, 60, 61, 62]. The NaAg_3_S_2_ and KAg_3_S_2_ data were obtained from experimental and computational results in this paper, respectively.

As discussed previously, low ν_*g*_ and short lifetimes (τ) typically result in reduced $\kappa_{L}$. Consequently, low $\kappa_{L}$ values are predominantly observed in compounds containing heavy elements,^[^ ^20^, ^52^, ^53^, ^54^ ^]^ such as Tl, Pb, and Bi. These heavier elements not only possess higher atomic masses but also have lower electronegativities, which enhances their propensity to form weaker chemical bonds. However, NaAg_3_S_2_‐type compounds exhibit a considerably lower average mass ($\bar{M}$) compared to other low‐$\kappa_{L}$ compounds, as illustrated in **Figure** **8**. Despite this, NaAg_3_S_2_ and KAg_3_S_2_ demonstrate significantly lower $\kappa_{L}$ values than other copper‐ and silver‐containing compounds with similar $\bar{M}$, making them competitive with compounds featuring heavier $\bar{M}$. It is noteworthy that Cu‐ and Ag‐containing compounds generally also exhibit low $\kappa_{L}$ values due to the presence of fully filled antibonding orbitals.^[^ ^25^ ^]^ As shown in Table S2 (Supporting Information), NaAg_3_S_2_‐type compounds possess relatively low ν_*g*_ (≈1500 m s^−1^), comparable to those of heavy $\bar{M}$ compounds such as Ag_8_SnSe_6_ and Ag_2_Te,^[^ ^53^ ^]^ despite their lower $\bar{M}$. This can be attributed to the weaker Ag‐S and Ag–Ag chemical bonds present in these compounds. Note that Ag_8_SnSe_6_ was recently found to exhibit extreme phonon anharmonicity and weak bonding, even at temperatures well below the superionic phase transition.^[^ ^15^ ^]^ Another consequence of these weak bonds is the strong anharmonicity, characterized by elevated phonon‐phonon scattering rates (τ^−1^), which includes both three‐phonon and four‐phonon scattering processes. Notably, the four‐phonon scattering rate in NaAg_3_S_2_ within the low‐frequency region is significantly larger than that of the three‐phonon scattering, which is typically dominant in phonon‐phonon interactions. Particularly, the four‐phonon scattering rates of the flat‐phonon bands in NaAg_3_S_2_ reach values as high as 10 ps^−1^, indicating strong anharmonicity that exceeds their vibrational frequencies and thus breaches the Ioffe–Regel limit. This leads to a substantial contribution to $\kappa_{L}$ from the off‐diagonal component, as illustrated in Figure 7b.

## 3 Conclusion

In this work, we proposed and demonstrated an effective strategy for weakening Ag–Ag chemical bonds within a Ag_6_ octahedron by enhancing the antibonding states below the Fermi level through the introducing an anion at the bridging position. Our material search based on this local coordination environment and new materials discovering based on structural decoration has yielded two existing and three synthesizable NaAg_3_S_2_‐type compounds. The combination of low sound velocity and strong anharmonicity, induced by the weak Ag–S and Ag–Ag chemical bonds, significantly reduces the $\kappa_{L}$ of NaAg_3_S_2_‐type compounds. The hardening of the flat‐band phonon modes associated with Ag atoms, along with the increased contribution from phonon tuning at elevated temperatures, results in a nearly temperature‐independent $\kappa_{L}$ across the temperature range of 200–550 K. Our experimental validation confirms that the $\kappa_{L}$ of NaAg_3_S_2_ is ≈0.45 W m^−1^ K^−1^ within the temperature range of 200–550 K. This finding underscores the effectiveness of our proposed material design strategy, which, founded on principles of chemical bonding, holds promise for application in other systems. The insights gained from this study not only advance our understanding of thermal transport mechanisms in low‐ and glass‐like $\kappa_{L}$ materials but also pave the way for the discovery of new compounds exhibiting desired thermal transport properties.

## 4 Experimental Section

### Computational Methods

The DFT calculations of lattice dynamics conducted in this work were carried out using the Vienna ab initio Simulation Package (VASP).^[^ ^63^, ^64^ ^]^ The projector augmented wave (PAW) method^[^ ^65^, ^66^ ^]^ with a plane wave basis set, employing a cutoff energy of 520 eV, was utilized. The calculations were performed using the PBEsol version^[^ ^67^ ^]^ of the generalized gradient approximation (GGA) exchange‐correlation functional. To sample the Brillouin zone, Γ‐centered *k*‐point grids were employed with a density exceeding 8000 *k*‐points per reciprocal atom (KPPRA). All structures were fully relaxed, with the forces on each atom constrained to be smaller than 0.01 eV Å^−1^, and the total energy convergence criterion was set to 10^−8^ eV. To elucidate the nature of chemical bonding, COHP analysis was performed for various pairs of nearest neighbor atoms using the LOBSTER code.^[^ ^68^ ^]^ The bulk modulus (*B*), shear moduli (*G*), and average speed of sound (ν_*a*_) were calculated from the elastic constants obtained through finite differences methods.

The second‐order force constants were calculated using the finite displacement method as implemented in the Phonopy code.^[^ ^69^ ^]^ A 2 × 2 × 2 supercell and a 2 × 2 × 2 *k*‐point mesh were utilized, with a displacement of 0.01 Å. The projection operators were evaluated in the reciprocal space (LREAL = FALSE) to enhance the accuracy of the force calculations. Random configurations were generated from 20 000 steps of ab initio molecular dynamics (AIMD) simulations based on machine learning force fields (MLFF),^[^ ^70^ ^]^ using a time step of 1 fs and a temperature of 300 K, from which 20 structures were selected. Self‐consistent calculations were subsequently performed to obtain displacement and force datasets. Additionally, compressive sensing lattice dynamics (CSLD)^[^ ^71^ ^]^ were employed to extract third‐ and fourth‐order force constants. The cutoff distances for the third‐ and fourth‐order interatomic force constants were limited to the sixth‐nearest and next‐nearest neighbors, respectively.

The anharmonically renormalized phonon frequencies at finite temperatures were calculated using SCPH theory.^[^ ^42^, ^43^, ^44^ ^]^ The thermal transport properties were obtained by solving the Peierls–Boltzmann transport equation through an iterative scheme implemented in the FourPhonon package.^[^ ^72^ ^]^ To ensure convergence, uniform 18 × 18 × 18 and 14 × 14 × 14 *q*‐point meshes were employed to calculate the contributions from SCPH three‐phonon scattering ($\kappa_{3\mathrm{ph}}^{P}$) and four‐phonon scattering ($\kappa_{3,4\mathrm{ph}}^{P}$), respectively. Additionally, the calculation of the four‐phonon scattering processes was accelerated using the sampling method.^[^ ^73^ ^]^ The off‐diagonal contributions from three‐phonon scattering ($\kappa_{3\mathrm{ph}}^{C}$) and four‐phonon scattering ($\kappa_{3,4\mathrm{ph}}^{C}$) were computed following the formalism developed by Simoncelli et al.^[^ ^8^ ^]^

### Experimental Methods

Polycrystalline samples of NaAg_3_S_2_ were synthesized using a melting and quenching technique. Stoichiometric amounts of sodium sulfide (Na_2_S, powder, 99.00%), silver (Ag, shot, 1–3 mm, 99.99%), and sulfur (S, pieces, 99.99%) were accurately weighed and placed into a graphite tube. This tube was subsequently loaded into a silica tube and sealed under a vacuum of ≈10^−4^ Pa. The sealed tube was heated to 1073 K over a period of 1000 min and maintained at this temperature for 1440 min, followed by rapid quenching in cold water. Given the brittleness of the sample, the resulting ingots were ground into fine powders and subsequently densified through hot‐pressing (HP) at 753 K for 15 min under a pressure of 30 MPa. The hot‐pressed pellet achieved a density of 96% of the theoretical density. The scanning electron microscopy (SEM) and energy‐dispersive spectroscopy (EDS) analyses show no distinct cracks or pores (see Figure S7, Supporting Information), confirming its highly densified nature.

Powder X‐ray diffraction (PXRD) of the hot‐pressed NaAg_3_S_2_ sample at room temperature was conducted using a Bruker D8 diffractometer, employing Cu K_α1_ radiation (λ = 1.5406 Å). Rietveld refinement was performed using the JANA20201 crystallographic computing system, based on the structural model reported by Huster et al.^[^ ^36^, ^74^ ^]^ As illustrated in Figure 1c, the Rietveld refinement of the PXRD pattern confirmed that the sample adopts the $Fd\bar{3}m$ (No. 227) crystal structure symmetry with a lattice parameter *a* = 12.3602(2) Å. The refined results, including atomic positions, isotropic atomic displacement parameters (U_iso_), atomic occupancy, and other related information, are tabulated in Table S3 (Supporting Information).

The thermal conductivity (κ) was calculated using the formula κ = ρC_*p*_ *d*. The thermal diffusivity (*d*) of the polycrystalline NaAg_3_S_2_ sample was assessed using a Netzsch LFA 457 laser flash system under a nitrogen atmosphere, spanning temperatures from 300 to 550 K. Low‐temperature measurements of κ and C_*p*_ below 300 K were obtained using a quantum design PPMS. The theoretical estimation of C_*p*_ above 300 K for NaAg_3_S_2_ is 0.36 Jg^−1^ K^−1^ according to the Dulong‐Petit approximation, and the density (ρ) was determined using the Archimedes method. The C_*p*_/T versus T^2^ plot (see Figure S3, Supporting Information) was fitted using the Debye–Einstein model, with the fitting parameters detailed in Table S4 (Supporting Information). Further discussion on this model and the fitting process can be found in the referenced work.^[^ ^13^ ^]^ The DSC and TGA were conducted from 300 to 600 K using a Netzsch STA 449F3 system. The SEM and EDS analyses were performed by a JEOL JSM 7200F scanning electron microscope.

## Conflict of Interest

All authors declare no competing interests.

## Supporting information

Supporting Information

## Data Availability

The data that support the findings of this study are available from the corresponding author upon reasonable request.
