# Supplementary material for: Realizing Intrinsically Ultralow and Glass‐Like Thermal Transport via Chemical Bonding Engineering
Source: Adv Sci (Weinh). 2025 Mar 7;12(17):2417292. doi: 10.1002/advs.202417292 (PMC12061320; doi:10.1002/advs.202417292)
Supplement: Supplementary file 1 — Supporting Information [file ADVS-12-2417292-s001.pdf]

ADVANCED SCIENCE
Open Access

Supporting Information

for *Adv. Sci.*, DOI 10.1002/advs.202417292

Realizing Intrinsically Ultralow and Glass-Like Thermal Transport via Chemical Bonding Engineering

Zhonghao Xia, Xingchen Shen, Jun Zhou, Yuling Huang, Yali Yang, Jiangang He* and Yi Xia**

Supplementary information of "Realizing Intrinsically Ultralow and Glass-like Thermal Transport via Chemical Bonding Engineering"

Zhonghao Xia,[†,#] Xingchen Shen,[*,‡,¶,#] Jun Zhou,[§] Yuling Huang,[‖] Yali Yang,[†] Jiangang He,[*,†] and Yi Xia[*,⊥]

†*Key Laboratory of Advanced Materials and Devices for Post-Moore Chips, Ministry of Education, School of Mathematics and Physics, University of Science and Technology Beijing, Beijing 100083, China*

‡*CRISMAT, CNRS,ENSICAEN,14000,Caen,France*

¶*MOE Key Laboratory of Material Physics and Chemistry Under Extraordinary Conditions, School of Physical Science and Technology, Northwestern Polytechnical University, Xi'an, 710072, People's Republic of China*

§*Basic Experimental Center for Natural Science, University of Science and Technology Beijing, Beijing 100083, China*

‖*Department of Mechanical and Energy Engineering, Southern University of Science and Technology (SUSTech), Shenzhen, 518055 China*

⊥*Department of Mechanical & Materials Engineering, Portland State University, Portland, OR 97201, USA*

#*Contributed equally to this work*

E-mail: xingchen.shen@nwpu.edu.cn; jghe2021@ustb.edu.cn; yixia@pdx.edu

Table S1: The energy difference (ΔE) between the $Fd\bar{3}m$ and the lowest-energy structures of AAg_3X_2 and the convex hull distance (E_h) of the AAg_3X_2 composition.

Comp.	Ag-X		Ag-Ag		A-X		E_h	ΔE
	bond length (Å)	-iCOHP	bond length (Å)	-iCOHP	bond length (Å)	-iCOHP	(meV/atom)	(meV/atom)
$Ag_3Ge_5P_6$	2.4903	1.1032	2.8426	0.5017	2.3268	4.7380	0	0
$LiAg_3S_2$	2.4681	1.3661	2.9773	0.1961	2.7151	0.5198	3	0
$NaAg_3S_2$	2.4418	1.5474	2.9760	0.1576	2.8989	0.4832	0	0
KAg_3S_2	2.4163	1.5728	2.9561	0.1299	3.1682	0.4040	4	24
$RbAg_3S_2$	2.4100	1.6142	2.9461	0.1275	3.3087	0.3672	0	44
$CsAg_3S_2$	2.4052	1.6441	2.9325	0.1245	3.4802	0.3126	56	60
$LiAg_3Se_2$	2.5680	1.3101	3.0291	0.1881	2.7903	0.5202	0	0
$NaAg_3Se_2$	2.5440	1.4484	3.0212	0.1717	2.9746	0.4876	0	6
KAg_3Se_2	2.5210	1.5215	2.9959	0.1617	3.2510	0.4179	33	60
$RbAg_3Se_2$	2.5161	1.5485	2.9792	0.1598	3.3963	0.3826	62	78
$CsAg_3Se_2$	2.5118	1.5456	2.9654	0.1509	3.5637	0.3388	25	93

Table S2: The fully relaxed lattice constants (a_{cal}) and average sound velocity (ν_{cal}).

Comp.	a_{cal} (Å)	ν_{cal} (m/s)
$Ag_3Ge_5P_6$	10.245	3045
$LiAg_3S_2$	11.776	1575
$NaAg_3S_2$	12.235	1479
KAg_3S_2	12.936	1924
$LiAg_3Se_2$	12.165	1489
$NaAg_3Se_2$	12.936	1436

Table S3: Refined crystallographic data of the hot-pressed $NaAg_3S_2$ powder at 300 K.

Atom	x	y	z	Uiso ($Å^2$)	Occ.
Na	0	0	0	0.0083(8)	1
Ag	0.125	0.125	0.4480(2)	0.0343(6)	1
S	0.2352(3)	0.2352(3)	0.2352(3)	0.0199(5)	1

Table S4: Fitting parameters of low-temperature C_p of the $NaAg_3S_2$ sample.

Compound	γ ($Jmol^{-1}K^{-2}$)	β ($10^{-4}Jmol^{-1}K^{-4}$)	Θ_D (K)	A_1 ($Jmol^{-1}K^{-1}$)	Θ_{E_1} (K)	A_2 ($Jmol^{-1}K^{-1}$)	Θ_{E_2} (K)	$v_{a,fit}$ (m/s)
$NaAg_3S_2$	0.0291(6)	4.23(5)	146	14.1(1)	30.1(1)	33.9(2)	65.4(2)	1322

Table S5: The elastic constants C_{ij} (GPa), bulk modulus B (GPa), Young's modulus E (GPa) and shear modulus G (GPa), Pugh's ratio B/G, Poisson's ratio ν, Debye temperature Θ_D (K), and minimal $\kappa_{\mathrm{L}}^{\mathrm{min}}$ ($\mathrm{Wm^{-1}K^{-1}}$).

Compound	C_{11}	C_{12}	C_{44}	B	E	G	B/G	ν	Θ_D	$\kappa_{\mathrm{L}}^{\mathrm{min}}$
$LiAg3S_2$	57.5	47.0	21.9	50.5	34.6	12.4	4.04	0.38	182	0.32
$NaAg_3S_2$	52.7	27.0	8.83	35.5	28.0	10.2	3.46	0.36	164	0.27
KAg_3S_2	84.4	28.8	13.1	47.3	47.6	17.8	2.65	0.33	213	0.33
$LiAg_3Se_2$	56.4	41.4	17.6	46.4	34.5	12.5	3.69	0.37	166	0.28
$NaAg_3Se_2$	49.1	22.2	9.38	31.2	29.1	10.8	2.87	0.34	154	0.24
$Ag_3Ge_5P_6$	113.2	59.1	52.6	77.1	102.9	40.2	1.91	0.278	338	0.46

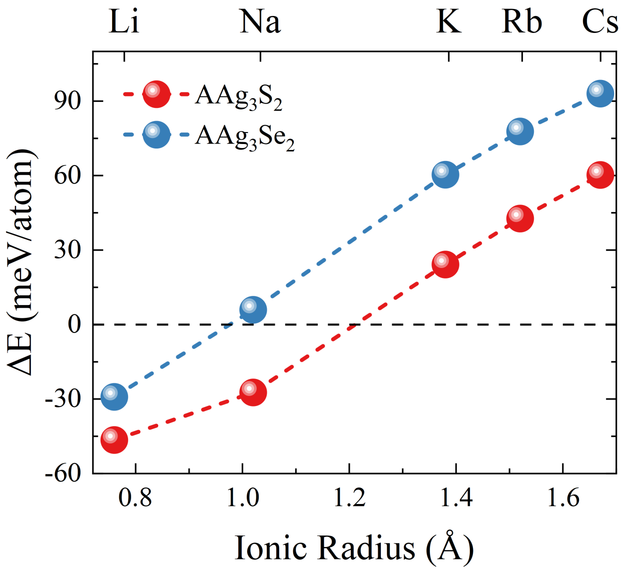

Figure S1: The energy difference (ΔE) between $Fd\bar{3}m$ and $C2/m$ for AAg_3X_2 compounds. Positive and negative ΔE indicate $C2/m$ and $Fd\bar{3}m$ are the ground state structure, respectively.

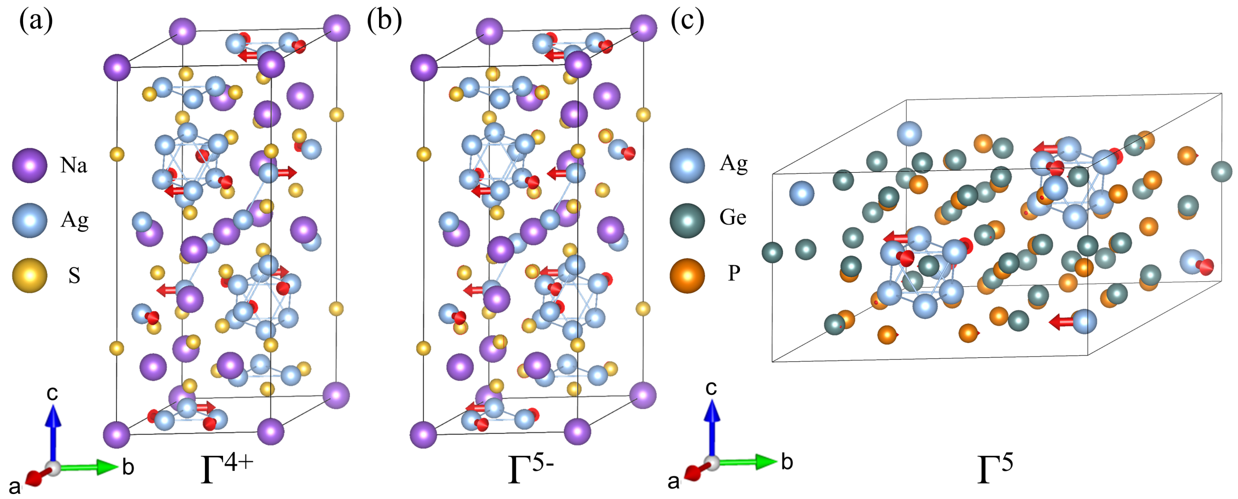

Figure S2: Atomic vibration at the Γ point corresponds to the (a) 4th branch and (b) 5th branch for $NaAg_3S_2$, (c) 5th branch for $Ag_3Ge_5P_6$, where the atomic displacement is indicated by red arrows while the light blue spheres represent Ag atoms.

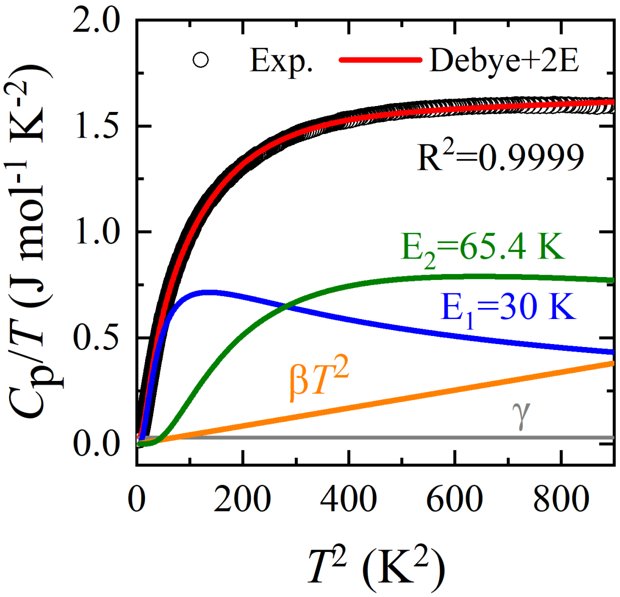

Figure S3: The fitting profile of the experimental data for C_p/T vs. T^2 with Debye-Einstein model.

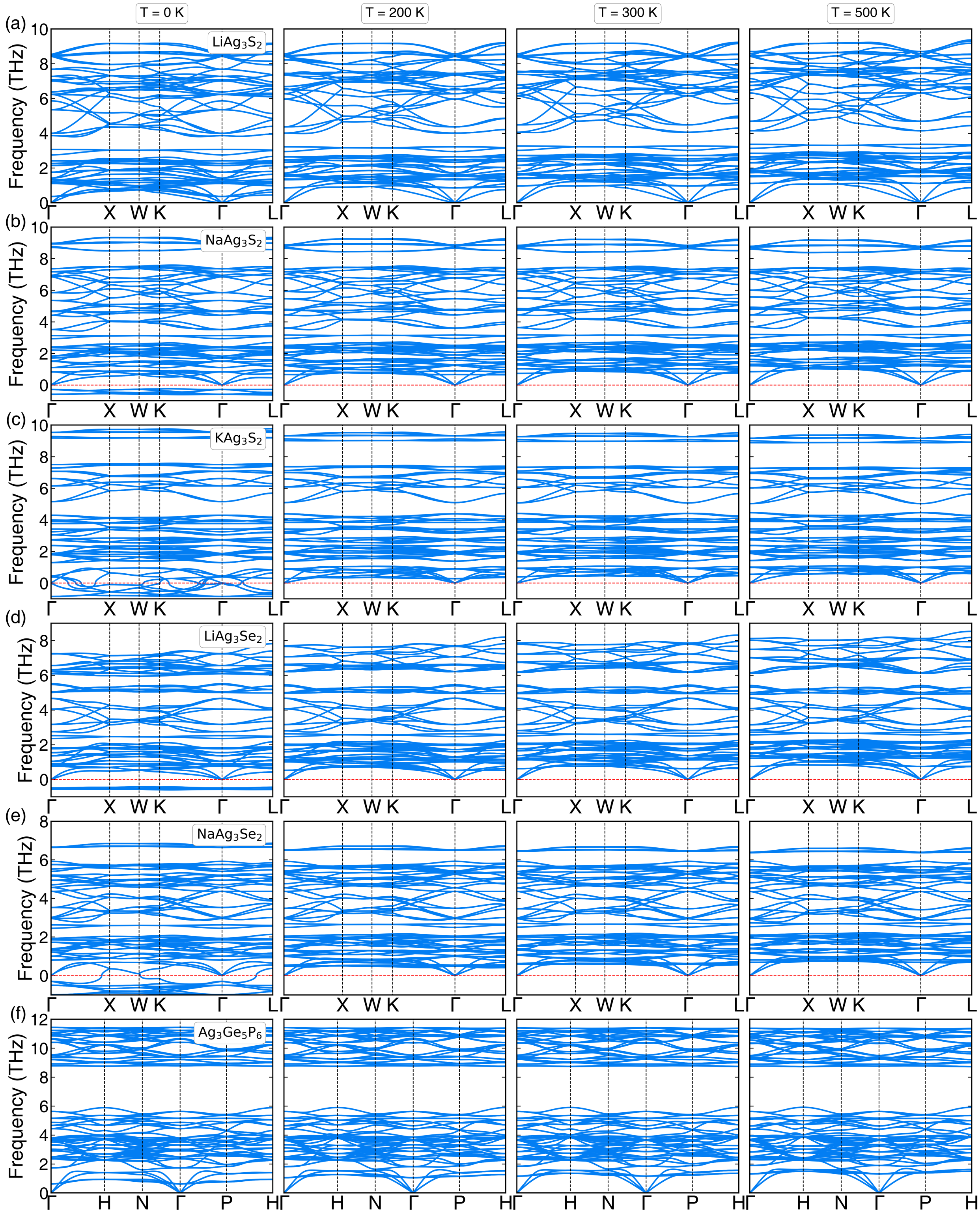

Figure S4: Calculated phonon dispersions of (a) $LiAg_3S_2$, (b) $NaAg_3S_2$, (c) KAg_3S_2, (d) $LiAg_3Se_2$, (e) $NaAg_3Se_2$, and (f) $Ag_3Ge_5P_6$ including anharmonic renormalization at finite temperatures (T = 200, 300, and 500 K) in comparison with those obtained from harmonic approximation.

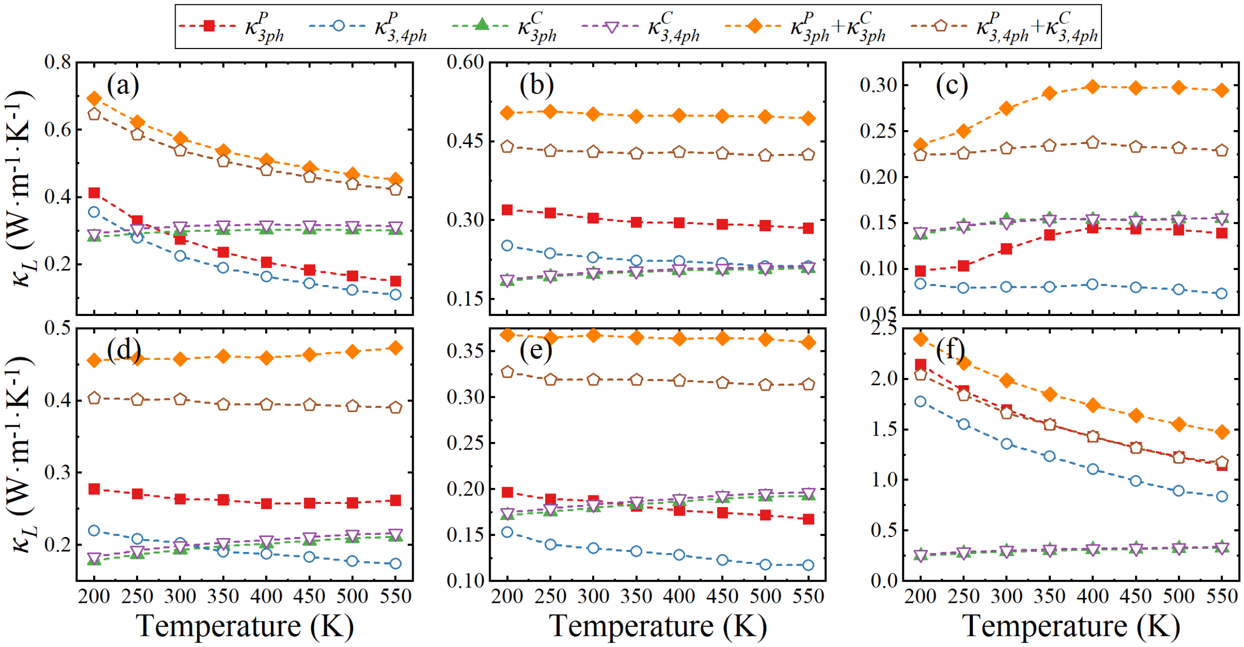

Figure S5: Calculated temperature-dependent κ_L considering only phonon populations' contribution and with additional coherences' contribution for (a) $LiAg_3S_2$, (b) $NaAg_3S_2$, (c) KAg_3S_2, (d) $LiAg_3Se_2$, (e) $NaAg_3Se_2$, and (f) $Ag_3Ge_5P_6$. The filled shapes denote the values obtained considering only three-phonon ($3ph$) scattering, whereas the empty shapes further include four-phonon ($4ph$) scattering.

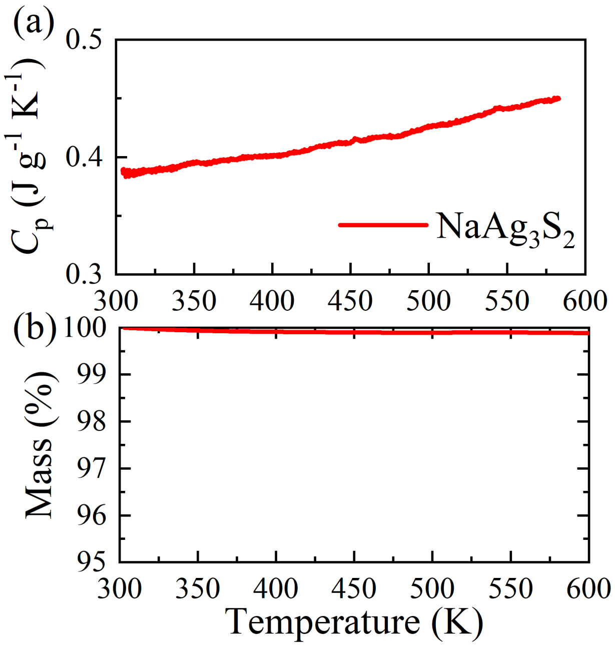

Figure S6: (a) The differential scanning calorimetry and (b) thermogravimetric analysis measurements of the $NaAg_3S_2$ sample from 300 to 600 K.

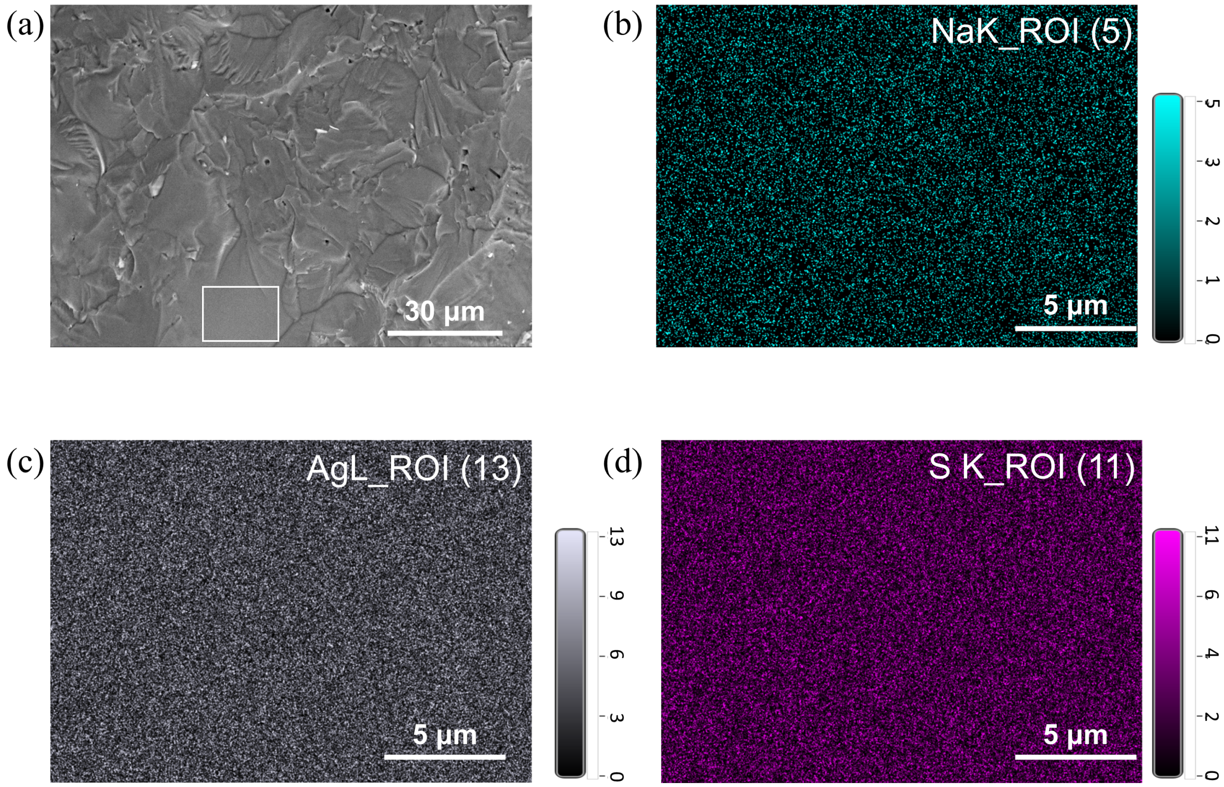

Figure S7: The SEM and EDS images of a fractured surface of the $NaAg_3S_2$ sample.

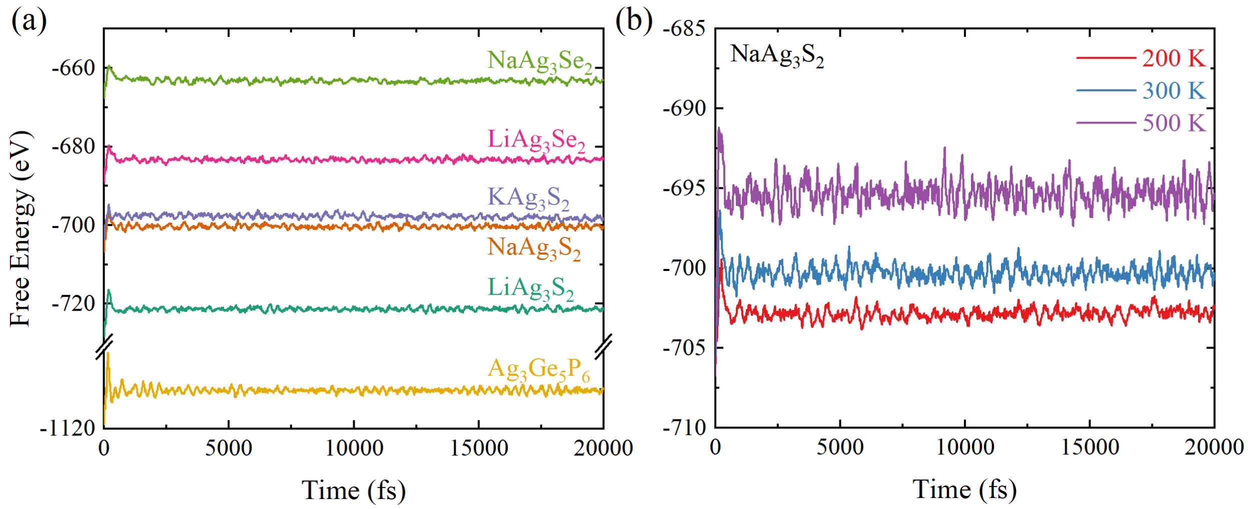

Figure S8: (a) The free energy as the function of time at 300 K for AAg_3X_2 composition. (b) The free energy as the function of time for $NaAg_3S_2$ for different temperature.
